# Developing a competence framework for gerontological nursing in China: a two-phase research design including a needs analysis and verification study

**DOI:** 10.1186/s12912-022-01074-y

**Published:** 2022-10-26

**Authors:** Bea L. Dijkman, Marina Hirjaba, Wenwen Wang, Marjo Palovaara, Marjolein Annen, Merle Varik, Ying’ai Cui, Jing Li, Cornelia van Slochteren, Wang Jihong, Chen Feiteng, Yu Luo, Yudong Chen, Wolter Paans

**Affiliations:** 1 Research Group Nursing Diagnostics, School of Nursing, Hanze University of Applied Sciences Groningen, Groningen, The Netherlands; 2 School of Health and Social Studies, JAMK University of Applied Sciences, Jyväskylä, Finland; 3 Nursing Department, Guangzhou Health Science College, Guangzhou, China; 4 Nursing and Midwifery Department, Tartu Health Care College, Tartu, Estonia; 5 School of Nursing, Guangdong Medical University, Dongguan, China; 6 Nursing College, Beihua University, Jilin, China

**Keywords:** Gerontological nursing, China, Competences, Nursing education, Competence framework

## Abstract

**Background:**

China faces a serious shortage of competent nurses who can address the healthcare needs of older people in an ageing society. Chinese higher education institutes face serious challenges when it comes to developing new curricula that are capable of educating sufficient numbers of competent gerontological nurses. Therefore, the aim of this research study was to identify and verify competencies for gerontological nurses in China that are needed to provide nursing care for the growing number of older people in all care settings. This study takes into account the possible opportunities that trends and developments may offer in the near future.

**Methods:**

In this study, a two-phase research design was used. The first phase concerned needs analysis, including a situational analysis, a trend analysis and a competence analysis. This process resulted in a draft competence framework. The second phase addressed the verification of the competence framework through a two-round Delphi study with a panel of Chinese and European experts. This process led to the final competence framework.

**Results:**

The final competence framework for gerontological nursing in China included six competencies divided into 13 essential and five relevant learning outcomes. The competencies are: ‘providing gerontological care’, ‘communication and collaboration’, ‘organization of gerontological nursing care’, ‘health promotion’, ‘evidence-based nursing and lifelong learning’ and ‘professional behaviour’.

**Conclusion:**

The framework comprehensively covers the six core competencies that nurses who care for older people should possess. These competencies are well-embedded in a Chinese context. The framework therefore offers concrete, practical suggestions for the competencies and skills that nursing graduates will need to work in current and future professions related to gerontological nursing education and practice.

**Supplementary Information:**

The online version contains supplementary material available at 10.1186/s12912-022-01074-y.

## Background

China faces a serious shortage of competent nurses who can address the healthcare needs of older people in an ageing society. According to the seventh population census of China’s National Bureau of Statistics, 264.02 million people in China are aged 60 or older, accounting for 18.70% of the country’s total population [1]. In contrast, there were 4.7 million registered nurses in China at the end of 2020, and the ratio of registered nurses per 1,000 people was 3.35 [2]. This is far below average international ratio, which is 8.8:1000 [3]. The Chinese government has offered various methods to increase the number of registered nurses and to meet the growing needs of older people. According to the National Health Commission of the People's Republic of China, increasing the number of registered nurses will require further efforts in healthcare education, healthcare organisations and healthcare infrastructure [4]. These changes are visible in the Healthy China 2030 Plan for the WHO [5] and in the country’s ongoing healthcare reform, which aims to increase training for medical professionals and to enhance investments in healthcare and care facilities for older people, especially in rural area [4]. Additionally, global cooperation and investments in new technologies are promoted to improve healthcare [4].

Gerontological care providers and educational institutes need to consider how to cope with the existing problems in the field of care for older people and how to address the development of gerontological nursing in China [6]. The Chinese nursing curriculum concerning gerontological nursing is quite traditional. From its inception, nursing education in China adopted the bio-medical model [7]. It focuses strongly on disease and theory, but it neglects prevention, health rehabilitation and practical skills [7, 8]. In general, graduates are neither motivated nor equipped with sufficient skills to work in the various healthcare environments that currently exist for older people [7]. Chinese higher education institutes (HEIs) face serious challenges when it comes to developing new curricula capable of educating sufficient numbers of competent gerontological nurses. Chinese nursing graduates need to acquire competencies to support healthy ageing today while also learning to understand the field’s future needs [7].

The GeNEdu project, which seeks to develop gerontological nursing education in China through multidisciplinary innovations, seeks to navigate the challenges in this field. In this Erasmus Capacity Building project, three Chinese HEIs work closely with three European HEIs. The main objective of the GeNEdu project is to renew nursing curricula by building the capacities of Chinese HEIs and by developing gerontological nursing education for future healthcare professionals to ensure that they can meet the needs of China’s ageing society [8]. Part of the project is the development of a competence framework for gerontological nurses in China. This framework is designed to guide the development of new educational programmes.

Competence frameworks typically are used to outline the characteristics of a competent workforce [9] and to serve as input for curriculum development [10]. In the field of healthcare, many competence frameworks exist, including several competence frameworks for gerontological nurses [11–13]. Most literature about gerontological nursing competences is based on research in western societies. China, in comparison to many other ageing societies, has distinctive national and cultural characteristics influencing gerontological nursing [8, 14]. Due to the cultural differences between China and Western countries that influence the perception of what gerontological care delivery entails, the need arose to develop a new competence framework as part of the GeNEdu-Erasmus project. Erasmus projects aim to bring expertise from European universities together with universities outside Europe, in order to learn from each other, conduct research together, and improve curricula [8]. GeNEdu’s competence framework for gerontological nursing will support the development of new curricula to meet the rising care needs of older people in China. Therefore, the aim of this research study was to identify and verify competencies for gerontological nurses in China.

## Methods

**Fig. 1:**
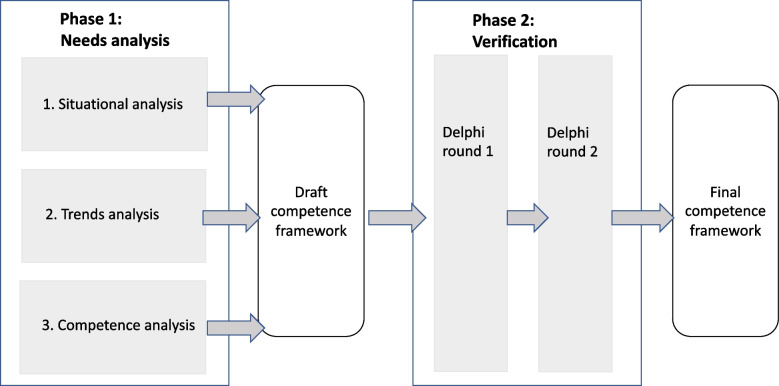
Two-phase research design, including needs analysis and verification study

In this study, a two-phase research design was used. The first phase concerned the needs analysis, including a situational analysis, a trend analysis and a competence analysis. It resulted in a draft competence framework. The second phase concerned the verification of the competence framework through a two-round Delphi study with a panel of Chinese and European experts. It led to the final competence framework (Fig. 1).

### Needs analysis

The design of the needs analysis included three steps. Its goal was to identify the competencies that gerontological nurses in China need. The first step of the needs analysis was a situational analysis, which identified the needs in gerontological nursing practice and education in China on both a national and regional level. The second step was a trends analysis, which identified the international trends and developments in gerontological nursing. The third step was a competence analysis, which provided insight into existing competence descriptions for gerontological nurses.

#### Situational analysis

Performing a situational analysis identified the primary challenges, trends and developments in gerontological care and gerontological nursing in China. A DESTEP analysis approach was used to identify the demographic, economic, social, technological, ecological and political factors that are important to the future of gerontological care and gerontological nursing education [15, 16]. During a one-day workshop, participants from three Chinese partner universities systematically mapped their findings onto national and regional levels. After the workshop, the Chinese partner universities performed desk research to deepen the DESTEP analysis. They used scientific articles, online information, policy documents and other relevant material written in Chinese. An additional six Chinese experts in the field of gerontological nursing practice were also interviewed. The outcomes of these interviews were shared and discussed during online meetings. This created mutual understanding about the current status and future needs of gerontological nursing in China.

#### Trends analysis

A comprehensive review of scientific and grey literature was performed to identify the main international trends in gerontological nursing and gerontological nursing education.

A scientific literature search was performed simultaneously in two databases: CINAHL and MEDLINE. The keywords used were ‘gerontology or elderly or aging or geriatric’ and ‘nursing or nursing care or nursing education or nursing competence’. The search was limited to peer-reviewed, full-text articles published from 2016–2020. They all addressed gerontological education and the care of older people in various settings. Additionally, a grey literature search was performed to discover handbooks, policy documents, educational material and other relevant material in the English or Finnish languages. Two researchers analysed the literature and independently listed the main trends (MH, MV). Rigour was addressed by discussing the findings with project team until a consensus was reached about the main trends present in gerontological nursing.

#### Competence analysis

The competence analysis was conducted to provide an overview of the existing core areas and competencies for gerontological nursing. A search was performed for documents that included competence descriptions relevant to gerontological nurses. The eligible documents included policy documents, professional reports, scientific articles and educational materials published after 2010 in Chinese, English, Finnish and Dutch. Materials were included until saturation was reached and no new competencies appeared.

The project members used a structured checklist to extract the relevant data, including competence descriptions, from the documents. BD performed a mapping exercise using an Excel spreadsheet to organise the competencies according to the seven CanMEDS roles [17]. The choice for CanMEDS roles was made on the basis of a consensus decision, which showed that the CanMEDS role model was widely known and recognized in nursing education. It also shows significant similarities with the competence framework of the European Federation of Nurses (EFN) [18]. This framework also has a good degree of implementation in various European countries.

After several face-to-face and online meetings among project members, consensus was reached about which competencies and learning outcomes to include in the draft competence framework. The results of the situational analysis and trend analysis were used as references to reduce the likelihood that important information would be missing from the draft competence framework.

### Verification

A Delphi panel with experts was consulted to verify the set of competencies for gerontological nurses in China. A Delphi study was chosen because this consensus-building tool has been applied in a variety of fields, including the development of competence frameworks [9, 19]. It is an appropriate method to use when experts are located at considerable geographic distances from each other, as was the case in this study. A Delphi study is predominantly qualitative in nature, but it also has a quantitative component whenever consensus is calculated [20].

#### The Delphi panel and procedure

A panel of experts was established by inviting 31 participants from China, Finland, Estonia and the Netherlands. Because the competencies should match the Chinese context, 75% of experts were selected from China. All experts were selected based on their authority and their high level of theoretical expertise in the field of gerontological nursing. Their professional backgrounds and settings included governmental organisations, long-term care facilities, community care providers and hospitals.

The Delphi study included two rounds of questionnaires concerning competencies and learning outcomes for gerontological nurses. The questionnaire for the first round was developed based on the results of the needs analysis. It included six competencies with a total of 21 learning outcomes. The questionnaire for the second round was composed based on the first round’s results. It included the competencies and learning outcomes that did not meet the consensus thresholds in the first round. Both questionnaires were translated from English to Chinese using the back-translation method [21]. The Chinese and English versions were distributed through Webropol.

Each participant received an individual link to the questionnaire via e-mail. They were all invited to rate the competencies and learning outcomes on a 3-point scale. For the first round, this scale included the following options: 1 (essential), 2 (useful but not essential) and 3 (not necessary) [22]. In addition, the questionnaire included open-ended questions to allow participants to provide additional comments. The 3-point scale in the second round included the following options: 1 (essential), 2 (very relevant) and 3 (relevant but not most important). No open-ended questions were included in the second round. Ratings were based on each participant’s personal opinions, and anonymity was guaranteed [23]. A reminder was sent after two weeks, and a second reminder was sent after four weeks.

#### Data analysis of the Delphi study

To analyse the first round’s ratings, content validity ratios (CVRs) were calculated. This is the appropriate technique to determine consensus among a panel of experts [22]. The CVR was determined as (*n*_e_—*N*/2)/(*N*/2), in which *n*_e_ is the number of panellists who indicated ‘essential’ and *N* is the total number of panellists. In a panel consisting of 25–30 people, a content validity ratio of 0.37 is regarded as the minimum value to define consensus [22].

In the second round of the questionnaire, competencies and learning outcomes with a CVR lower than 0.37 and a percentage higher than 50% were presented for validation again. If the percentage was lower than 50%, it was considered to be nonessential, and it was removed from the competence framework after discussing the interpretation of the data with the project team. Open answers provided evidence for changes in the formulation of the competencies and learning outcomes.

## Results

### Needs analysis

#### Situational analysis

The situational analysis resulted in a summary of the specific characteristics that define the care of older people and the practice of gerontological nursing in China. Many older people live alone; these so-called "empty nesters" account for more than 54% of the country’s older population [24]. This group of people receives limited care and support – or none at all – from their family members. A large gap exists in the quality of life and poverty levels between the urban and rural populations. There are more women than men, and, especially in rural China, older women face great disadvantages in every aspect of life.

At present, gerontological nursing in China faces certain problems. These include a serious workforce shortage, the low payment of professional nurses in gerontological nursing and a lack of care institutions for older people. In addition, many nurses working in the gerontological care have lower education levels, and they may lack skills in technology or in other areas of contemporary nursing.

An important aspect of nursing practice is reflected in the Chinese cultural understanding of health and the use of traditional Chinese medicine (TCM) interventions to restore the overall balance, rather than simply treating the symptoms [25]. Experts agree that TCM should be integrated in future care as well because older people in China benefit from it. To obtain all-round development, nursing students should acquire basic theory, knowledge and skills of TCM [26].

Innovative models for gerontological care and the use of technology are needed to improve the quality of services for elderly people. China promotes new modes of nursing services, such as "smart elderly care" which is based on a sensor network system and information platform for elderly at home, communities and care organizations [27].

#### Trends analysis

The scientific database search located 117 scientific articles. After screening by title, abstract and full text, 14 scientific articles were included for analysis. In addition, 22 documents were selected from the grey literature search.

Content analysis revealed five trends in gerontological nursing. The first is an emphasis on person-centred care, which relates to the needs of older people and their families [28, 29]. Nurses should recognize individual and cultural factors and provide person-centred care in innovative ways. The second trend is a willingness to collaborate with and to account for the needs of family members and informal caregivers. Because family members often become part of the healthcare team, nurses require skills and knowledge to involve family caregivers accordingly [30–32]. The third trend in gerontological nursing is a focus on health promotion and healthy ageing via disease prevention and a healthy lifestyle [28, 33]. The fourth trend is support for independent living at home; many older people remain at home and use technological solutions in gerontology [34–36]. The fifth trend is multidisciplinary collaboration, which is important when a patient has multiple health or social issues. Complex, multifactorial interventions can significantly improve older adults’ ability to remain living at home and avoid residential care admission [37, 38].

It is possible to identify trends towards improved education programmes and the use thereof, particularly in settings such as home care, community care, long-term care and end-of-life care. Home care includes health maintenance, counselling and education to prevent illness; it also includes disease treatment, rehabilitation and palliative care [31]. Community nursing might be a cost-efficient way to decrease the burden of informal caregivers and primary care providers [39]. In long-term care settings, gerontological nurses ought to have the competence to recognize factors that contribute to a better quality of life for residents [31, 40, 41]. Additionally, nurses in these settings should emphasize their competence in pharmacotherapy [42]. For quality end-of-life care, a structured and evidence-based educational programme is recommended to nursing staff [30, 43]. End-of-life care provision must be possible in settings such as hospitals, homes or community and rehabilitation placements [44]. Nurses and health team members provide the medical, emotional and spiritual support needed in end-of-life care [34, 44]. Because HEIs educate the future workforce, it is important to incorporate these trends into nursing curricula.

#### Competence analysis and the draft competence framework

In total, 14 Chinese policy documents, 17 competence frameworks, 32 scientific articles and four educational materials were included in the competence analysis (see Table 1 an Appendix 1). The policy documents covered different fields of work for gerontological nurses in China. Competence descriptions in these policy documents focused primarily on competencies that belong to the expert or manager roles. The 17 competence frameworks included competencies covering all seven CanMEDS roles. Eight of the frameworks explicitly mentioned technology as part of the competencies. Additional competencies were retrieved from the scientific articles and the educational materials, which covered all seven CanMEDS roles.

**Table 1:** Number of documents included in the competence analysis—breakdown per region

	China	European countries	USA/Australia/ New Zealand	Asian countries	Other/ International	Total
Policy documents	14	0	0	0	0	14
Competence frameworks	1	7	9	0	0	17
Scientific articles	23	4	3	2	0	32
Curriculum descriptions	1	2	0	0	1	4

Synthesis of the competence descriptions resulted in a draft competence framework of gerontological nursing. Its six competencies were: ‘providing gerontological care’, ‘communication and collaboration’, ‘nursing leadership and innovation’, ‘health promotion’, ‘evidence-based nursing and lifelong learning’ and ‘professional behaviour’. The six competencies were elaborated via 21 learning outcomes. For the purpose of the GeNeDU competence framework, competencies were defined as a dynamic combination of knowledge, skills, attitudes and values capable of being transferred to a certain context or real situation. Learning outcomes were defined as statements about what a learner is expected to know, understand and be able to demonstrate after the completion of learning [45].

### Results verification phase

#### Participants’ characteristics

The first round included 29 participants, and the second round involved 26. The majority of them had more than 10 years of work experience and a nursing background. The majority were working at healthcare organizations when they answered the questionnaires (see Table 2). Both Delphi rounds identified the same background characteristics among participants.

**Table 2:** Delphi participant characteristics (*n* = 26)

		*n*	%
**Gender**	Female	22	85%
	Male	4	15%
**Age**	30–39 years	1	4%
	40–49 years	8	31%
	50–59 years	10	38%
	> 59 years	7	27%
**Years of work experience in gerontological care**	< 5 years	0	0%
	5–10 years	8	31%
	> 10 years	18	69%
**Current work**	University	8	31%
	Healthcare organization	12	46%
	Other	6	23%
**Profession**	Teacher	8	31%
	Researcher	7	27%
	Nurse	12	46%
	Management member at university	1	4%
	Management member at health care organization	10	38%
	Other	5	19%
**Field of expertise in gerontological nursing**	Home care	10	38%
	Hospital care	12	46%
	Long-term care	12	46%
	Other	2	8%

### Delphi study

After the first round of the Delphi study, a high level of consensus was found for the competencies ‘providing gerontological care’, ‘communication and collaboration’ and ‘professional behaviour’ (see Table 3). The experts agreed that these three competencies and the learning outcomes associated with them are essential for gerontological nurses in China. The CVR score for these three competencies was 0.93. The CVR scores for the corresponding learning outcomes varied from 0.45–0.86.

**Table 3:** The Delphi study’s first round

**Competences & learning outcomes**	**Essential**			**Useful**			**Can be missed**		
	**%**	**n**	cvr*	**%**	**n**	**cvr**	**%**	**n**	**cvr**
**Competence: Providing gerontological care**	96.55%	28	0.93	3.45%	1	-0.93	0%	0	-1.00
a) Assessment	89.66%	26	0.79	10.34%	3	-0.79	0%	0	-1.00
b) Nursing diagnosis	79.31%	23	0.59	20.69%	6	-0.59	0%	0	-1.00
c) Planning	93.1%	27	0.86	6.9%	2	-0.86	0%	0	-1.00
d) Implementation of nursing interventions	89.66%	26	0.79	10.34%	3	-0.79	0%	0	-1.00
e) Evaluation	86.21%	25	0.72	13.79%	4	-0.72	0%	0	-1.00
**Competence: Communication and collaboration**	96.55%	28	0.93	3.45%	1	-0.93	0%	0	-1.00
a) Patient centered communication and empowerment	86.21%	25	0.72	13.79%	4	-0.72	0%	0	-1.00
b) Collaborate with family members and informal caregivers	72.41%	21	0.45	24.14%	7	-0.52	3.45%	1	-0.93
c) Collaborate with nursing colleagues and the multidisciplinary team	86.21%	25	0.72	13.79%	4	-0.72	0%	0	-1.00
**Competence: Nursing leadership and innovation**	65.52%	19	**0.31**	34.48%	10	-0.31	0%	0	-1.00
a) Leadership	48.27%	14	**-0.03**	48.28%	14	-0.03	3.45%	1	-0.93
b) Planning and coordination of care and services	65.52%	19	**0.31**	34.48%	10	-0.31	0%	0	-1.00
c) Policy development	37.93%	11	**-0.24**	58.62%	17	0.17	3.45%	1	-0.93
d) Innovation and technology	58.62%	17	**0.17**	41.38%	12	-0.17	0%	0	-1.00
e) Quality management	82.76%	24	0.66	17.24%	5	-0.66	0%	0	-1.00
**Competence: Health promotion**	79.31%	23	0.59	17.24%	5	-0.66	3.45%	1	-0.93
a) Develop health promotion interventions	62.07%	18	**0.24**	37.93%	11	-0.24	0%	0	-1.00
b) Advocate for older people	44.83%	13	**-0.10**	51.72%	15	0.03	3.45%	1	-0.93
c) Social map and social networks	44.83%	13	**-0.10**	51.72%	15	0.03	3.45%	1	-0.93
**Competence: Evidence based nursing and lifelong learning**	68.97%	20	0.38	31.03%	9	-0.38	0%	0	-1.00
a) Life long learning and professional development	72.41%	21	0.45	27.59%	8	-0.45	0%	0	-1.00
**b) Critical thinking and evidence based practice**	65.52%	19	**0.31**	31.03%	9	-0.38	3.45%	1	-0.93
c) Training and coaching	75.86%	22	0.52	20.69%	6	-0.59	3.45%	1	-0.93
**Competence: Professional behaviour**	96.55%	28	0.93	3.45%	1	-0.93	0%	0	-1.00
a) Professional ethics	93.1%	27	0.86	6.9%	2	-0.86	0%	0	-1.00
b) Professional commitment and personal awareness	75.86%	22	0.52	24.14%	7	-0.52	0%	0	-1.00

^*^Total *n* = 29, cvr threshold = 0.37

The competence ‘nursing leadership and innovation’ showed a CVR of 0.31. Analysing the ratings according to the level of the learning outcomes associated with this competence showed that the learning outcome ‘quality management’ was the only one that experts agreed to be essential. With a CVR of 0.31, the learning outcome ‘planning and organisation’ was close to the threshold value. The other learning outcomes – ‘leadership’, ‘policy development’ and ‘innovation and technology’ – showed CVR scores between -0.24 and 0.17. Although the competence ‘health promotion’ was rated as essential, there was no consensus about the three learning outcomes associated with this competence; they had CVR scores between -0.10 and 0.24. For the competence ‘evidence-based nursing’, only the learning outcome ‘evidence-based nursing and critical thinking’ was below the threshold for determining consensus; it had a CVR of 0.31. Because the CVR could not determine a consensus regarding the essentiality of the three competencies ‘nursing leadership and innovation’, ‘health promotion’ and ‘evidence-based nursing and lifelong learning’, these competences were reformulated and presented to the panel of experts in the second round of the Delphi study. The reformulation of these competencies and learning outcomes was based on the CVR scores and remarks that participants made.

Table 4 presents the second round’s scores. The competence ‘nursing leadership and innovation’ was reformulated towards ‘organisation of gerontological nursing care’, which resulted in higher ratings for its essentiality. The reformulated learning outcome ‘innovation and technology’ was still not considered to be essential, but it was rated very relevant. For the competence ‘health promotion’, the learning outcome ‘plan person-centred health promotion’ was rated essential. ‘Perform health promotion’ was rated below the CVR threshold for essentiality. Therefore, it was considered very relevant.

**Table 4:** The Delphi study’s second round

	**Essential (cannot be missed)**			**Very relevant**			**Relevant but not most important**		
	**%**	**n**	cvr*	**%**	**n**	**cvr**	**%**	**n**	**cvr**
**Competence: Organisation of gerontological nursing care**	80.8%	21	0.62	19.2%	5	-0.62	0.0%	0	-1.00
a) Planning and coordination of care and services	88.5%	23	0.77	11.5%	3	-0.77	0.0%	0	-1.00
b) Innovation and technology	46.2%	12	-**0.08**	50.0%	13	0.00	3.8%	1	-0.92
e) Quality management	73.1%	19	0.46	19.2%	5	-0.62	7.7%	2	-0.85
**Competence: Health promotion**	73.1%	19	0.46	26.9%	7	-0.46	0.0%	0	-1.00
a) Plan person-centered health promotion	73.1%	19	0.46	23.1%	6	-0.54	3.8%	1	-0.92
b) Perform health promotion interventions	61.5%	16	**0.23**	34.6%	9	-0.31	3.9%	1	-0.92
**Competence: Evidence based nursing and lifelong learning**	61.5%	16	**0.23**	34.6%	9	-0.31	3.9%	1	-0.92
a) Life long learning and professional development	73.1%	19	0.46	26.9%	7	-0.46	0.0%	0	-1.00
b) Evidence based practice	57.7%	15	**0.15**	30.8%	8	-0.38	11.5%	3	-0.77
c) Training and coaching	50.0%	13	**0.00**	46.2%	12	-0.08	3.8%	1	-0.92

^*^n = 26, cvr threshold = 0.37

The competence ‘evidence-based nursing and lifelong learning’ shows a diffuse pattern. In the first round, this competence and two of the three associated learning outcomes were considered to be essential. Only the learning outcome ‘evidence-based nursing and critical thinking’ was below the consensus threshold. In the second round, there only seemed to be consensus that the learning outcome ‘lifelong learning and professional development’ was essential. The other learning outcomes were considered very relevant.

The Delphi study resulted in a verified final competence framework that showed the essential and relevant competencies and learning outcomes (see Table 5). The competence "providing gerontological care" and the corresponding learning outcomes describe care and planning of care on the level on one individual patient. The competence "organisation of gerontological care" and the corresponding learning outcomes concern the organisational level.

**Table 5:** Final competence framework: six core competences and 18 learning outcomes

**1. PROVIDING GERONTOLOGICAL CARE**	***Importance***
**Competence: providing gerontological care** The gerontological nurse comprehensively assesses, analyses, plans, implements and evaluates the care of older people. The gerontological nurse is able to utilize evidence-based knowledge and critical thinking when making decisions and providing person-centred and holistic care in different care settings. The nurse considers the wishes and physical and mental well-being of older people and their families by supporting all parties’ active participation.	***Essential***
a. **Assessment** Conduct a systematic, comprehensive gerontological assessment with input from the older people and, when necessary, from their families or caregivers. Inquire about the older people’s physical and mental well-being, medical history, personal history, housing conditions, social participation and loneliness. Identify the needs, wishes and possibilities to increase older people’s comfort. Assess the level of nursing needs.	***Essential***
b. **Nursing diagnosis** Analyse the data collected from the gerontological assessment and, through careful consideration, form a diagnosis using knowledge about healthy ageing, geriatric syndromes and the most common health problems among older people. Identify the problems and risk factors for older people and their families. Diagnose the required nursing care using current theoretical and clinical knowledge of the nursing process.	***Essential***
c. **Planning** Develop a clear, timely and appropriate plan for person-centred nursing care with a focus on recovery, optimal health, well-being and quality of life for older people and their families. Use practice- and evidence-based interventions. If possible, include the use of technology for the benefit of the patient and family members. Use appropriate techniques for shared decision-making.	***Essential***
d. **Implementation of nursing interventions** Provide accurate implementation of the care plan and perform the nursing interventions based on professional nursing standards in different care settings such as home care, hospital care, long-term care and hospice care. Guarantee person-centred and holistic care.	***Essential***
e. **Evaluation** Evaluate and adjust care plans for older people on a continuing basis with the purpose of providing optimal nursing care for older people and their families.	***Essential***
**2. COMMUNICATION AND COLLABORATION**	
**Competence: communication and collaboration** To provide person-centred care, the gerontological nurse communicates and collaborates with older people, family members, other informal caregivers and other professionals in health and social care. The nurse is able to use Information and Communication Technology (ICT) properly for this purpose.	***Essential***
a. **Person-centred communication and empowerment** Form strong, positive professional relationships with older people based on empathy, trust, respect and reciprocity. Communicate in a clear and effective way considering older people’s individuality, sociocultural backgrounds, health problems and needs. Collaborate with patients, use shared decision-making and empower older people to take responsibility for their own health and comfort.	***Essential***
b. **Collaborate with family members and informal caregivers** Work together with older people’s supportive families, informal caregivers and social networks to encourage appropriate informal care and support. Be aware of older patients who suffer from loneliness and family members who suffer from caregiver burden.	***Essential***
c. **Collaborate with nursing colleagues and the multidisciplinary team** Work effectively together with other professionals for integrated care and support. Encourage multi- and inter-professional cooperation to achieve optimal support and care for older people. Pursue the goal of optimising their health, well-being and quality of life in multiple areas.	***Essential***
**3. ORGANISATION OF GERONTOLOGICAL NURSING CARE**	
**Competence: organisation of gerontological nursing care** The nurse plans and coordinates safe, high-quality person-centred care for older people. The nurse is involved in quality assurance activities and contributes to the innovation of care for older people; this includes the use of suitable technical applications in care.	***Essential***
a. **Planning and coordination of care and services** Plan, arrange and coordinate the care and services provided by nurses and other formal or informal health and social care workers across different organizations to provide the best personalized care and support for older people and their families. Ensure continuity of care.	***Essential***
b. **Innovation and technology** Use innovative ideas, theories and methods to improve gerontological nursing practice. This process includes the use of technological applications.	***Relevant***
c. **Quality management** Initiate, monitor and participate in quality management activities to provide safe, high-quality person-centred nursing care for older people. Establish assessment mechanisms and processes for continuous quality improvement.	***Essential***
**4. HEALTH PROMOTION**	
**Competence: health promotion** The gerontological nurse is able to prevent further functional decline and to promote healthy ageing and a healthy lifestyle. The nurse helps older people and their families find comprehensive person-centred solutions within the entire healthcare system.	***Essential***
a. **Plan person-centred health promotion** Identify early risk factors that can impact the functional ability of older people. Plan holistic and person-centred health promotion interventions.	***Essential***
b. **Perform health promotion interventions** Work closely together in partnerships with patients, informal caregivers and other healthcare professionals to promote a healthy lifestyle and to work towards the improved self-care of older people.	***Relevant***
**5. EVIDENCE-BASED NURSING AND LIFELONG LEARNING**	
**Competence: evidence-based nursing and lifelong learning** The gerontological nurse uses evidence-based practices and lifelong learning activities to provide the best care for older people and their families.	***Relevant***
a. **Lifelong learning and professional development** Increase knowledge, understanding and skills in gerontological nursing through continuous education and professional development. Demonstrate commitment to lifelong learning.	***Essential***
b. **Evidence-based practice** Use and support the implementation of evidence-based nursing’s theory and methodology in gerontological care.	***Relevant***
c. **Training and coaching** Participate as a teacher and coach in education and training activities about gerontological nursing for staff, students and teachers. Strengthen the competencies of nursing staff in gerontological nursing.	***Relevant***
**6. PROFESSIONAL BEHAVIOUR**	
**Competence: professional behaviour** The gerontological nurse shows a professional attitude, is aware of professional guidelines and is committed to providing appropriate person-centred care for older people and their families.	***Essential***
a. **Professional ethics** Provide nursing care for older people in accordance with professional and personal ethics, legal guidelines and cultural sensitivities.	***Essential***
b. **Professional commitment and personal awareness** Demonstrate commitment to providing appropriate gerontological nursing care for older people and their families. Be aware of personal values and assumptions influencing professional practice. Act within professional frameworks and legislation.	***Essential***

## Discussion

The gerontological nursing competence framework for China includes six competencies and 18 learning outcomes. This framework comprehensively covers the core competencies that nurses who care for older people should possess. One strength of this comprehensive competence framework is its applicability to different care settings. The competence descriptions focus on the current and future needs of gerontological nursing.

We distinguished 13 essential and five relevant learning outcomes. The essential learning outcomes are all closely related to providing nursing care for patients and their family members. The learning outcomes that were rated as relevant are more supportive and more closely related to the organisation of care, innovation, personal development and professional development. Huizinga et al. (2016) explained that the more distant from patient activities a role is, the less frequently competencies are considered to be essential [46]. This includes competencies about social networks; research and innovation of care; legal, financial and organizational issues; professional ethics; and professional innovation [46]. Experts rated the learning outcome "Advocate for older people" as irrelevant for gerontological nurses in China, due to cultural and social values [14]. The competence framework targets vocational and bachelor levels. It includes the competencies related to organisation, leadership, research and innovation that are of foremost importance for bachelor-level students [18]. Although experts did not rate these competencies as essential, they should be part of gerontological education at a bachelor level.

The method used, in which Chinese gerontological nursing experts were involved in the needs analysis and the verification process, resulted in a competence framework that is well embedded in a Chinese context [14]. Although the competencies are specifically designed for gerontological nursing in China, the wording of the competencies and their associated learning outcomes is quite general. The GeNedu project used the results of the needs analysis to develop a handbook with more detailed descriptions of the required knowledge and assessment criteria for the learning outcomes that include specific cultural elements [8]. For example, the GeNEdu competence framework’s competencies and learning outcomes do not mention TCM, which is considered to be important for nursing practice in China. In the handbook TCM knowledge and skills are integrated in the competence "providing gerontological care" as part of holistic care. We recommend to take this into account during the curriculum development process, while specifying learning outcomes and developing educational content for the competencies associated with gerontological care.

The use of technology is becoming increasingly important for gerontological nurses. The competence framework’s learning outcome ‘innovation and technology’ shows this importance, but it is also embedded in all of the competencies. Further elaboration for educational purposes should include the key areas where digital technology is needed to provide high-quality, ethical patient care; social and communication skills; diagnoses and treatment; motivation and willingness to integrate digitalisation in a professional context; and collegial and organisational support for building positive experiences via digitalisation [47].

As most of the literature about gerontological nursing competences is based on research in western societies, our study focused on the transfer and application in Chinese culture. This is as far as we know the first study that compared and integrated information from different cultures in this way. In addition, the method we used may also be of interest to project leaders of similar curriculum development projects in other countries.

The Delphi method was suitable to reach consensus among the experts. Because of the extensive needs analysis, two rounds were sufficient [48]. One strong point of our approach is the feedback it provided to experts during the second round. It could be considered a limitation that we used a different scale during the second round of the Delphi study. On the other hand, this method provided deeper nuance in the competence set by distinguishing essential competencies from competencies that were relevant, but not essential.

### Implications for practice

In China, this competence framework, which was developed through a process of international cooperation, will challenge Chinese institutions to meet international standards regarding the quality of nursing in higher education. The competence framework is available in Chinese and English. It will be a useful instrument for developing future gerontological nursing curricula in China. It offers concrete, practical suggestions about the competencies and skills that nursing graduates need for current and future gerontological nursing practice.

In general, the use of the competence framework will require a shift from theory-based to competence-based education. Training is recommended to help teachers develop competence-based education and to make learning outcomes specific, operable and comparative. Within the GeNEdu project, Chinese teachers who participated in such a training, developed successfully six modules for a gerontological nursing curriculum.

Since teaching and assessing learning outcomes is new to Chinese teachers, sustainable implementation of these modules will require training of all teachers. Additionally, the core competences for educators in gerontological nursing can help the Chinese institutes to develop these teacher trainings [49].

## Conclusion

The gerontological nursing framework for China includes six competencies with 13 essential and five relevant learning outcomes. The competences are ‘providing gerontological care’, ‘communication and collaboration’, ‘organization of gerontological nursing care’, ‘health promotion’, ‘evidence-based nursing and lifelong learning’, and ‘professional behaviour’. The framework comprehensively covers the core competencies that nurses who care for older people should possess. These competencies are well embedded in a Chinese context.

## Supplementary Information

**Additional file 1.**

## Data Availability

The datasets used and analysed during the present study are available from the corresponding authors upon reasonable request.

## Abbreviations

CVR: Content validity ratio
HEI: Higher educational institute
ICT: Information and communication technology
WHO: World Health Organization
