# Supplementary material for: Developing a competence framework for gerontological nursing in China: a two-phase research design including a needs analysis and verification study
Source: BMC Nurs. 2022 Oct 26;21:285. doi: 10.1186/s12912-022-01074-y (PMC9597998; doi:10.1186/s12912-022-01074-y)
Supplement: Supplementary file 1 — Additional file 1. [file 12912_2022_1074_MOESM1_ESM.pdf]

GeNEdu COMPETENCES GERONTOLOGICAL NURSING: DOCUMENT ANALYSIS

	Title	Country	year		description of trends and developments	competences	learning outcomes	knowledge	skills	attitudes	other.........	nursing in general	gerontological nursing	working with older people in health and social car	0 other, ...	hospital care for older person	long term care organisations/facilities	home care	other.....	not specified (national nurse)	vocational level (EFQ 4/5)	bachelor level (EFQ 6)	master level (EFQ 7)	other, ...	Expert	Communicator	Collaborator	Manager	Health Advocate	Scholar	Professional	Techonolgy
	**POLICY DOCUMENTS**																															
1	National nursing development Plan(2016-2020)	China	2016		1							1																1				
2	Notice on boosting care for the elderly	China	2019		1											1	1															
3	Opinions of the General Office of the State Council on Setting up and Carrying out the Projects for Elderly Care Services	China	2017			1		1	1				1	1		1	1	1		1												
4	Notice on carrying out needs assessment and	China	2019			1			1				1	1					1						1			1				
5	standardizing service of elderly care																															
6	Guiding opinions on promoting the construction of livable environment for the elderly	China	2016			1		1	1				1			1	1	1		1												
7	Notice on accelerating the construction of health and pension service project	China	2014		1									1			1				1	1			1			1				
8	Notice on printing and distributing “the 13th Five-year plan” for healthy aging	China	2017		1								1	1																		
9	Notice of the general office of the National Health Commission on the implementation the psychological care project for the elderly	China	2019					1	1					1			1								1			1				
10	Long term care insurance service standards and norms	China	2019		1							1																				
11	Seven experiences and 23 typical cases of the reform of home-based and community-based elderly care service in China	China	2019		1								1					1														
12	Training curriculum for gerontological nursing	China	2019					1	1				1			1	1															
13	New Standards for Gerontological Care: "Notice on Carrying out the Needs Evaluation and Standardization of Elderly Care"	China	2019		1								1			1	1	1	1	1					1			1				

14	National Occupational Skills Standards for Elderly Caregivers	China	2019						1					1					1		1											
	**COMPETENCE FRAMEWORKS**																															
1	European Federation of Nurses Competency Framework 2015.	Europe	2015		1	1	1	1	1			1	1	1		1	1	1		1		1			1	1	1	1	1	1	1	1
2	Nurse's Professional Competences. The Future of Nursing Education Project.	Finland	2015				1					1				1	1	1				1			1	1	1	1	1	1	1	1
3	National Competence requirements and contents (180 ECTS credits) for general nurse education in Finland. Development Project for National examination for nurses in Finland.	Finland	2020			1			1			1				1	1	1				1										
4	Report of Competence Needs in Elderly Care.	finland	2013		1	1	1						1	1			1	1	1		1	1	1	1	1	1	1	1		1	1	1
5	Bachelor of Nursing 2020	The Netherlands	2018			1		1	1	1	1	1	1							1		1			1	1	1	1	1	1	1	1
6	Expertisegebied Geriatrieverpleegkundige	The Netherlands	2016			1							1			1	1	1			1	1			1	1	1	1	1		1	
7	Developing an Inter-disciplinary Cross-Setting Dementia Care Competency Framework	Australia	2015			1							1			1	1	1	1	1					1	1	1	1	1		1	
8	Gerontology NursingKnowledge and Skills Framework 2014	New Zealand	2012			1	1	1	1				1				1					1			1	1	1	1	1		1	1
9	Gerontological Nursing – competencies for care	USA	2010			1		1	1	1			1			1	1	1				1	1		1	1	1	1	1	1	1	
10	Multidisciplinary Competencies in the Care of Older Adults at the Completion of the Entry-level Health Professional Degree	USA	2008			1								1											1	1	1	1	1			
11	Recommended Baccalaureate Competencies and Curricular Guidelines for the Nursing Care of Older Adults.	USA	2010			1			1				1			1	1	1	1			1			1	1	1	1	1			1
12	The Pikes Peak Model Competencies: Attitude, Knowledge, and Skill Competencies for Practice in Professional Geropsychology	USA	2009			1		1	1	1					1					1		1	1	1								
13	Multidisciplinary Competencies in the Care of Older Adults at the Completion of the Entry-level Health Professional Degree	USA	2010			1								1						1					1	1	1	1	1	1	1	1
14	Gerontology competencies for undergraduate & graduate education	USA	2014			1							1							1		1	1		1	1	1	1	1	1	1	
15	Nurse Practitioner and Clinical Nurse Specialist Competencies for Older Adult Care	USA	2004			1									1																	
16	European Core competnce Framework working with older people in health and social care	Europe	2017			1		1	1	1				1		1	1	1				1			1	1	1	1	1	1	1	1
17	Training program for nurses in specialized nursing	China				1		1	1			1			1													1				
	**SCIENTIFIC ARTICLES**			**First author**																												

1	Older people's views and expectations about the competences of health and social care professionals: a European qualitative study.	Europe	2019	Soares		1			1	1				1		1	1	1								1	1				1	
2	Emotional competencies in geriatric nursing: empirical evidence from a computer based large scale assessment calibration study.	Germany	2016	Kaspar		1								1		1	1	1			1	1										
3	Substituting physicians with nurse practitioners, physician assistants or nurses in nursing homes: a realist evaluation case study	Netherlands	2019	Lovink														1														
4	Learnt and perceived professional roles of a new type of nurse specialized in Gerontology and Geriatrics, a qualitative study	Netherlands	2016	Huizinga		1							1			1	1	1				1			1	1	1	1	1	1	1	
5	Developing gerontological nursing competencies: An e-Delphi study.	Australia	2018	Britten		1							1												1	1	1	1	1	1	1	1
6	Gerontology competencies: Construction, consensus and contribution	USA	2019	Damron-Rodriguez		1							1												1	1	1	1	1	1	1	
7	Development of core competencies and a recognition program for gerontological nursing educators	USA	2019	Wymen		1							1												1		1	1		1	1	1
8	Emotional competence: A core competence in gerontological nursing in Iran	Iran	2018	Bahrami		1							1			1								1							1	1
9	The Components of Nursing Competence in Caring for Older People in Iranian Hospitals: A Qualitative Study.	Iran	2019	Bahrami		1							1			1									1		1		1	1		
10	The Competence Standards of Nursing Degree Programs in Europe and the Enlightenment-	China	2020	Li Hui		1							1								1	1	1		1	1	1	1	1	1	1	
11	The Development Goals of Nursing Care in China and the Development Project of Nursing Care Services in the 13th Five-Year Plan Period	China	2017		1								1	1											1			1				
12	Research on the core competence framework of registered nurses	China	2011	Xu Shaobo		1							1					1							1	1	1	1		1	1	
13	Revision and verification analysis of Geriatric Nursing Competency Inventory	China	2016	GUO Jiajia		1							1							1												
14	Research on the Cultivation of Entrepreneurship Capability of the Elderly Nursing Specialty in Higher Vocational Colleges under the "Internet +"	China	2019	Zhu Xinxin		1							1								1									1		
15	Construction of Core Competence Evaluation Index System for Undergraduate Gerontological Nursing Talents	China	2016	Liu Yue		1							1									1										
16	Current Status of Geriatric Nursing Competence of Clinical Nurses in Rate A Tertiary Hospital in Guangdong Province and Related Influencing Factors	China	2019	Liang Hao		1							1			1								1	1	1		1		1	1	

17	Construction of Evaluation Indexes of Core Competence for Geriatric Specialized Nurses	China	2016	Liu Dunxiu		1							1							1												
18	Preliminary Construction of Evaluation Indicators for Continuing Nursing Core Competence of Geriatric Nurses	China	2019	Jiang Jingjing		1							1			1								1								
19	Survey of Needs of Senior Citizens in Medical Care Pension Agency Core Competence of Aged Care	China	2019	Chen Juan		1							1				1															
20	Development of Gerontology Community Nurse Competencies	China	2017	Cao Jing		1							1						1	1									1		1	
21	The Research on the Situation Analysis and Promotion Effect of Elderly Nursing Professional Competence of Secondary Vocational and Technical School Students	China	2018	Kuang Wenjing	1	1							1								1										1	
22	Construction of Competence Evaluation System for Gerontological Nurses	China	2017	Li Yuzhi		1							1											1	1	1	1		1	1	1	
23	Survey and Analysis of Knowledge and Core Competence Needs of Gerontological Nursing Talents in Yunnan Province	China	2017	Zhao Jie	1	1							1			1	1	1	1					1	1	1	1	1	1	1	1	
24	Preliminary Construction of Core Competency Framework for Nurses under Framework of Multisited Practice	China	2019	Yu Siping		1							1						1						1		1		1	1	1	
25	Construction of the Training Model for the Gerontological Senior Practical Nurses Based on Core Competence	China	2018	Jiang Jiaxiang		1							1			1									1		1	1		1	1	
26	Initial Study on Core Competency Framework for Higher Vocational Student Nurses on Job Requirements	China	2017	Hu Min		1							1			1					1				1	1	1				1	
27	Construction of the Evaluation Index System for the Core Competence of Medical-Nursing Combined Nursing Professional and Its Reliability and Validity Test	China	2020	Xi Yangjuan		1							1			1									1	1	1	1	1	1	1	
28	Development and Psychometric Testing of the Caregiver Communication Competence Scale in Patients With Dementia.	China	2015	Chao							1			1			1									1						
29	Analysis on the connotation, index and framework Of nursing core competence and core competency in China	China	2011			1				1											1	1	1							1		
30	A study on gerontological nursing ability index system of professional nurses	China				1	1		1				1			1	1	1				1	1									
31	Instrument Development for Evaluation of Gerontological Nurse Specialists Core Competencies in China	China	2019	Chen				1	1	1			1											1	1	1	1	1	1	1	1	
32	Student nurses' career motivation toward gerontological nursing: A longitudinal study	China	2019	Chai								1									1	1										

	**CURRICULUM DESCRIPTIONS**																															
1	JAMK online education education module for nurses “Multicultural Gerontological Nursing” (5 ECTS)	Finland				1	1	1	1				1			1	1	1	1			1										
2	Guide to Core Competence of Professional Nurses	China				1						1				1				1												
3	European Master's Program in Gerontology (EuMaG): Goals, Curriculum, and Students	International																														
4	WHO Europe Gerontological Nursing Curriculum	International			1	1							1			1						1			1	1	1	1	1	1	1	
